# Identification of a Cryptic Prokaryotic Promoter within the cDNA Encoding the 5′ End of Dengue Virus RNA Genome

**DOI:** 10.1371/journal.pone.0018197

**Published:** 2011-03-31

**Authors:** Dongsheng Li, John Aaskov, William B. Lott

**Affiliations:** Infectious Diseases Program, Institute of Health and Biomedical Innovation (IHBI), Queensland University of Technology, Brisbane, Australia; Duke-National University of Singapore Graduate Medical School, Singapore

## Abstract

Infectious cDNA clones of RNA viruses are important research tools, but flavivirus cDNA clones have proven difficult to assemble and propagate in bacteria. This has been attributed to genetic instability and/or host cell toxicity, however the mechanism leading to these difficulties has not been fully elucidated. Here we identify and characterize an efficient cryptic bacterial promoter in the cDNA encoding the dengue virus (DENV) 5′ UTR. Following cryptic transcription in *E. coli*, protein expression initiated at a conserved in-frame AUG that is downstream from the authentic DENV initiation codon, yielding a DENV polyprotein fragment that was truncated at the N-terminus. A more complete understanding of constitutive viral protein expression in *E. coli* might help explain the cloning and propagation difficulties generally observed with flavivirus cDNA.

## Introduction

Flaviviruses are single-stranded positive-sense RNA viruses belonging to the family *Flaviviridae*. These arthropod-borne viruses are responsible for a wide range of diseases, including Japanese encephalitis, yellow fever and tick-borne encephalitis. Dengue viruses (DENV) are the causative agents of dengue fever (DF), dengue hemorrhagic fever (DHF) and dengue shock syndrome (DSS) [1], [2]. Approximately 50–100 million DENV infections occur each year [2], resulting in 250,000 cases of DHF/DSS. The development of an effective DENV vaccine strategy has been difficult, because there is only short-lived cross-protection among the four DENV serotypes, and antibody dependent enhancement of DENV infections leads to the more severe forms of the disease [3], [4]. Full-length infectious cDNA clones of all four DENV serotypes would greatly enhance the development of tetravalent DENV vaccines.

As with other RNA viruses, flavivirus genome-length infectious cDNA clones are important tools for the genetic analysis of virus replication and pathogenesis, drug design and vaccine development. However, constructing and propagating flavivirus cDNA infectious clones in bacteria has proven difficult [5], presumably due to host cell toxicity and/or genetic instability [6]. To overcome these difficulties, flavivirus cDNA clones have been prepared in low copy number plasmids, bacterial artificial chromosomes, yeast plasmids, or by propagating sub-genomic fragments that could be assembled *in vitro* to produce complete genomes [5], [7], [8], [9], [10]. Even successful flavivirus infectious cDNA clones constructed in low-copy number plasmids remain deleterious to *E. coli*, leading to slow growth and poor DNA yields [5]. Stabilizing spontaneous nonsense mutations were often found in the regions encoding structural proteins in constructs containing the cDNA of Japanese encephalitis virus (JEV) [11]. The genetic instability of JEV cDNA clones has been attributed to unintentional transcription from phage or bacterial promoters in the vectors used. Similarly, spontaneous genetic rearrangements were observed in *E. coli* when cDNA encoding the 5′ fragment of the West Nile virus (WNV) genome was cloned in the direct orientation downstream from bacterial promoters, but not in the opposite orientation, suggesting that the characteristic flavivirus genetic instability was caused by viral gene product toxicity to the bacterial cells [12].

Expression of toxic viral gene products presumably requires unexpected transcription in *E. coli*, yet no bacterial promoter activity from within the cloning vectors or the viral cDNA itself has been identified. In this study, we identify and characterise a cryptic bacterial promoter within the cDNA encoding the dengue virus (DENV) 5′ untranslated region (UTR). In a reporter construct, this promoter leads to the efficient expression of a DENV polyprotein fragment fused to the green fluorescent protein (GFP). To our knowledge, this is the first report of a specific cryptic promoter within flavivirus cDNA. An efficient cryptic promoter in the DENV cDNA, which might be a common feature among mosquito borne flaviviruses, might help explain the difficulty of establishing infectious DENV cDNA clones.

## Results

### 5′ DENV2 cDNA sequence drives cryptic expression of viral protein in *E coli*

**Figure 1:**
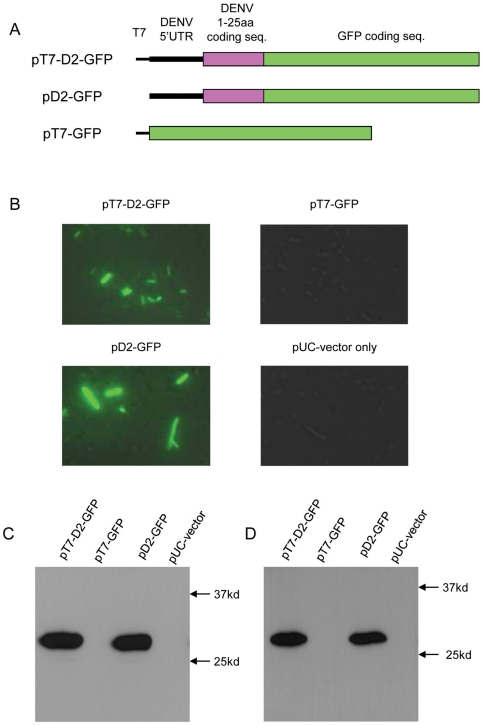
Expression of D2-GFP fusion protein in *E. coli* (DH5α) is driven by a cryptic promoter in the cDNA encoding the 5′ 1–170 nt of DENV2 RNA genome. (A) Schematic for the constructs of pT7-D2-GFP, pD2-GFP and pT7-GFP. (B) Florescence microscopy images of *E. coli* cells transformed with these plasmids. (C) Western blot analyses of transformed *E. coli* lysates employing 6F3.1 anti-dengue 2 virus core protein monoclonal antibody. (D) Western blot analyses of transformed *E. coli* lysates employing the anti-GFP antibody.

The plasmid pT7-D2-GFP was constructed by inserting a T7 promoter sequence, cDNA encoding the 5′ 1–170 nt of DENV serotype 2 (DENV2) and the GFP sequence into a pUC18 vector (Figure 1A). The DENV2 cDNA sequence includes the complete 5′ UTR (DENV nt 1–95) and the 5′ portion of the DENV2 open reading frame that codes for the N-terminal 25 amino acids of the capsid protein. The GFP sequence was cloned in frame with the authentic DENV2 start codon. Thus, mRNA transcribed from this construct would be expected to produce a fusion protein in eukaryotic cells consisting of the N-terminal 25 amino acids of the DENV2 polyprotein fused to GFP. Compenent DH5α *E. coli* cells (Promega) transformed with pT7-D2-GFP fluoresced under UV illumination in the absence of IPTG induction. To control for strain variation, these transformations were repeated using MAX Efficiency® Stabl2™ competent cells (Invitrogen). No difference in protein expression was observed between cell types (data not shown). Therefore, only data produced from the DH5α strain are reported for these and subsequent transformation experiments. As the pUC18 vector did not contain a constituent bacterial promoter, this observation suggested that the cDNA encoding the 5′ end of the DENV genome contained an efficient cryptic prokaryotic transcriptional promoter.

To evaluate this hypothesis, two plasmids based on pT7-D2-GFP were constructed. The DENV2 cDNA sequence was deleted in pT7-GFP, while the T7 promoter sequence was deleted in pD2-GFP. *E. coli* transformed with plasmids containing the DENV2 cDNA sequence (pT7-D2-GFP or pD2-GFP) fluoresced strongly, while cells transformed with plasmids lacking this sequence (pT7-GFP or the pUC18 vector-only control) did not fluoresce (Figure 1B). These data show that the expression of GFP was not due to leaky transcription by the T7 promoter or from unexpected promoter activity in the vector itself, and that the DENV2 sequence is responsible for the observed GFP expression. To confirm that the GFP fluorescence arose from the expression of the expected fusion protein, D2-GFP, proteins from lysates of transformed *E. coli* were resolved by SDS-PAGE, blotted, and probed with either a monoclonal antibody that recognised the DENV capsid protein [13] (Figure 1C) or one that recognised GFP (Figure 1D). Both antibodies recognised a protein of about 28 kDa in lysates of *E. coli* cells transformed with either pT7-D2-GFP or pD2-GFP, while no proteins were detected in lysates of cells transformed with plasmids lacking the DENV2 cDNA sequence (pT7-GFP or pUC vector). These data suggested that a cryptic transcriptional promoter in the 5′ 170 nt of DENV2 cDNA led to the efficient expression of an authentic DENV2 protein sequence in *E. coli*. Although there was no evidence of leaky T7 promoter activity, subsequent experiments used constructs that lacked a T7 promoter unless *in vitro* transcription was required.

### A cryptic prokaryotic promoter is located in the cDNA encoding DENV2 nt 68–86, and the resulting mRNA does not require a Shine-Dalgarno sequence for translation initiation

**Figure 2:**
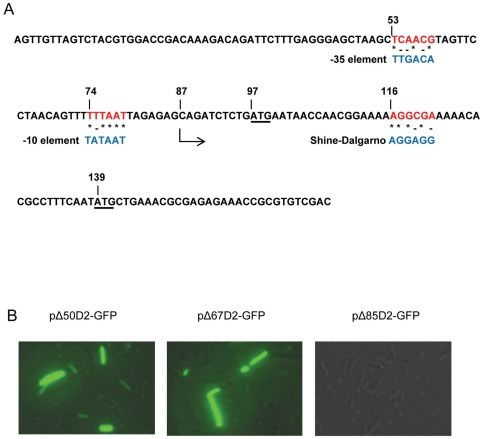
Cryptic promoter sequence analysis. (A) The cDNA sequence encoding the 5′ terminal 170 nt of the DENV2 RNA genome. The putative −10 and −35 cryptic promoter elements and the putative Shine-Dalgarno sequences (red) are aligned with their corresponding *E. coli* wild type elements (blue). The cDNA encoding the authentic DENV2 start codon (97AUG) and the in-frame alternate start codon (139AUG) are underlined. The predicted transcription initiation site is at the cDNA encoding DENV2 nt 87. (B) Fluorescent microscopy images of *E. coli* cells transformed with deletion mutant plasmids that result in truncations of 50 nt, 67 nt and 85 nt from the 5′ end of DENV2 RNA, respectively.

The BPROM promoter prediction program (SoftBerry, Mount Krisco, NY) identified potential −35 and −10 bacterial promoter elements at DENV2 cDNA nt positions 53 (TCAACG) and 72 (TTTTTAAT), respectively, which share sequence homology with the wild type *E. coli* promoter elements (Figure 2A). All four DENV serotypes contain similar, but not identical, sequences in this T-rich region. Based on these predictions, the start of cryptic transcription should be at or about DENV2 cDNA nt position 87, which is 10 nt upstream from the authentic DENV2 start codon (97AUG). Attempts to use 5′ RACE to locate the transcriptional start site more precisely were unsuccessful.

To determine whether the putative cryptic promoter elements were functioning in *E. coli*, 50, 67 or 85 bp were removed from the 5′ end of the DENV2 cDNA in pD2-GFP to produce the plasmids pΔ50D2-GFP, pΔ67D2-GFP and pΔ85D2-GFP, respectively. The predicted −35 and −10 elements were both present in the parental plasmid (pD2-GFP) and in pΔ50D2-GFP, while the −35 element was absent in pΔ67D2-GFP, and both the −35 and the −10 elements were absent in pΔ85D2-GFP. Deleting the first 50 bp or 67 bp of DENV2 cDNA had no effect on D2-GFP expression in transformed *E. coli*, while deleting bp 1–85 abolished D2-GFP expression altogether (Figure 2B).

To characterise the role that transcription played in this observation, the amount of GFP mRNA produced in *E. coli* was determined by quantitative RT-PCR (Table 1). *E. coli* transformed with pΔ50D2-GFP and pΔ67D2-GFP yielded approximately the same amount of GFP mRNA as *E. coli* transformed with the parental pD2-GFP plasmid. By contrast, the GFP mRNA yield in *E. coli* transformed with pΔ85D2-GFP was reduced by three orders of magnitude relative to the parental plasmid. Thus, the putative −10 element of the cryptic promoter sequence appeared to be essential for function, while the putative −35 element did not.

**Table 1:** Quantitative detection of GFP gene in total cellular RNA extracted from *E. coli* cells transformed with pD2-GFP, pD2-GFP mutants and pKUN-GFP.

Plasmid	Copies of mRNA*	P value
pD2-GFP	6.6	
pΔ50D2-GFP	6.5	>0.05
pΔ67D2-GFP	6.3	>0.05
pΔ85D2-GFP	3.8	<0.01
pD2-74G-GFP	5.7	<0.05
pD2-74GCG-GFP	4.6	<0.01
pKUN-GFP	5.7	<0.05

*Log_10_ copies of GFP gene/µg of total RNA.

To determine if the efficiency of cryptic transcription correlated with sequence within the DENV2 nt 68–86 region, the presumed −10 element (DENV2 nt 72–79) in pD2-GFP was mutated, and the yields of GFP mRNA produced in *E. coli* transformed with these plasmid constructs were assayed by quantitative RT-PCR (Table 1). The yield of GFP mRNA was reduced by an order of magnitude when the wild type −10 element, TTTTTAAT (pD2-GFP), was mutated to TTGTTAAT (pD2-74G-GFP), and by two orders of magnitude when it was mutated to TTGCGAAT (pD2-74GCG-GFP). Such modulation of transcription efficiency is consistent with a cryptic bacterial promoter in this region.

**Figure 3:**
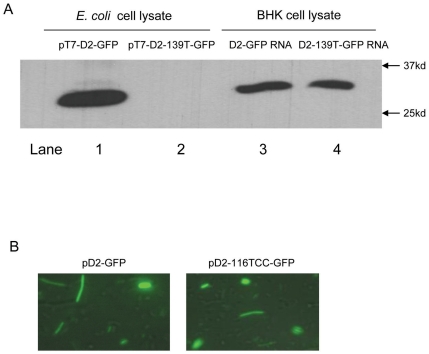
D2-GFP translation initiates in *E coli* at an alternate start codon (139AUG) by a Shine-Dalgarno independent mechanism. (A) Western blots of *E. coli* (lanes 1 and 2) and BHK (lanes 3 and 4) cell lysates expressing D2-GFP and D2-139T-GFP probed with anti-GFP antibodies. *E. coli* was transformed with either pT7-D2-GFP or pT7-D2-139T-GFP. BHK cells were transfected with *in vitro* transcribed D2-GFP and D2-139T-GFP RNA. (B) Fluorescent microscopy images of *E. Coli* cells transformed with either pT7-D2-GFP or pT7-D2-116TCC-GFP.

To compare the expression of D2-GFP in bacterial cells and in eukaryotic cells, D2-GFP RNA was transcribed from pT7-D2-GFP *in vitro* and transfected into BHK cells. Expression of D2-GFP was assessed by Western blot analyses of lysates of either transformed *E. coli* or RNA-transfected BHK cells. The molecular weight of the fusion protein expressed in BHK cells, estimated by electrophoretic mobility to be about 30 kDa, was approximately 1.5 kDa larger than the fusion protein expressed in *E. coli* (Figure 3A, compare lanes 1 and 3), suggesting that translation had initiated in *E. coli* downstream from the initiation site employed in BHK cells. DENV2 normally initiates translation in eukaryotes by a scanning mechanism [14], [15] at the first AUG encountered on the mRNA (97AUG, Figure 2A). Initiation from the AUG at nt 139 (139AUG) of the DENV2 RNA, which is in frame with 97AUG, would yield a D2-GFP protein of a size consistent with the 28 kD band observed in the western blot of *E. coli* lysates. To determine if 139AUG was used to initiate translation in *E. coli*, an A139T mutation was introduced into pT7-D2-GFP that would convert the 139AUG methionine (Met) codon to a 139UUG leucine codon in the resulting mRNA. BHK cells transfected with RNA transcribed from pT7-D2-GFP-139T expressed a D2-GFP fusion protein of the same size as the protein expressed in BHK cells that had been transfected with RNA from pT7-D2-GFP (Figure 3A, compare lanes 3 and 4). However, a D2-GFP fusion protein was not observed in *E. coli* transformed with this plasmid (pT7-D2-GFP-139T) (Figure 3A, lane 2), confirming that translation in *E. coli* required the 139AUG.

Since both 97AUG and 139AUG are predicted to be present in mRNA transcribed by the DENV2 cryptic promoter, 139AUG is likely to be in a more favourable context than 97AUG for translation initiation in bacteria. While there is no wild type Shine-Dalgarno (SD) sequence encoded in the 5′ DENV2 cDNA, the 116–121 nt (AGGCGA) sequence is the most similar (Figure 2A). To determine if this region functions as a SD sequence, the AGG at DENV2 cDNA nt 116–118 in pD2-GFP was mutated to TCC (pD2-116TCC-GFP). *E. coli* transformed with either pD2-GFP or pD2-116TCC-GFP fluoresced strongly under UV illumination (Figure 3B), indicating that the DENV2 nt 116–118 is not required for bacterial translation.

### Constitutive transcription from the 5′ DENV2 cDNA cryptic promoter leads to efficient protein expression in *E. coli* in the absence of IPTG induction

**Figure 4:**
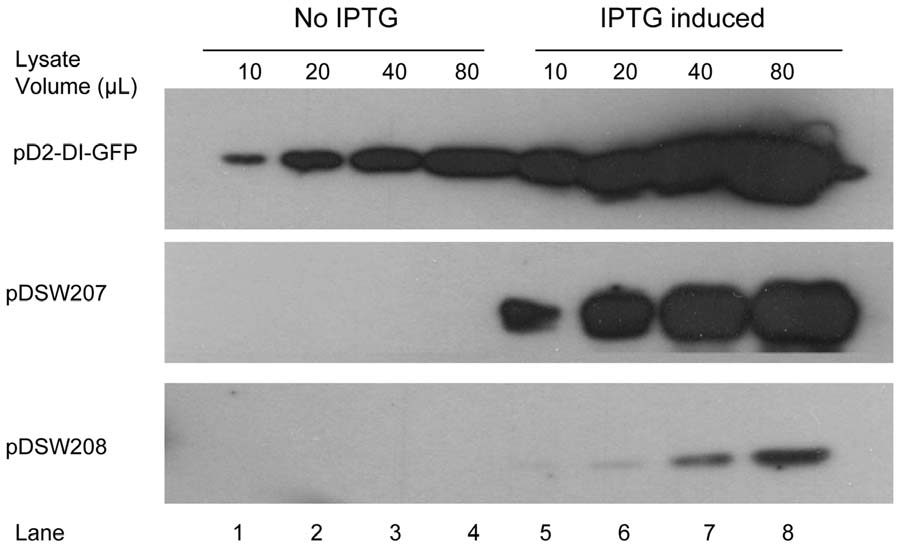
The efficiency of the DENV cDNA cryptic bacterial promoter. *E coli* cells transformed with pD2-GFP, pDSW207 or pDSW208 were cultured at 37°C for 5h in the absence (lanes 1–4) or presence (lanes 5–8) of IPTG. The numbers of bacterial cells in each culture were normalized, and the cells were pelleted by centrifugation before lysis. Proteins were resolved by SDS-PAGE and analysed by Western blot employing anti-GFP antibodies. The volume of clarified lysate loaded into each lane is shown.

To assess the efficiency of the 5′ DENV2 cDNA cryptic promoter sequence, pD2-GFP expression in *E. coli* was compared with two well-characterized GFP expression constructs, pDSW207 and pDSW208 (17), which promote GFP expression in *E. coli* using modified *trc* promoters that are relatively strong and weak inducible promoters, respectively. In the absence of IPTG, the D2-GFP fusion protein was efficiently expressed in *E. coli* transformed with pD2-GFP, while no GFP expression was detected in cells transformed with either pDSW207 or pDSW208 (Figure 4). When induced with IPTG, D2-GFP protein expression in *E. coli* transformed with pD2-GFP under the control of the pUC18 *lac* promoter increased approximately 6 to 8 fold relative to uninduced expression, and was similar to IPTG-induced pDSW207 GFP expression. Constitutively expressed D2-GFP from pD2-GFP was more efficient than IPTG induced GFP expression from pDSW208 in *E. coli*.

### Kunjin virus contains a similar but less efficient cryptic bacterial promoter in the cDNA encoding its 5′ UTR

**Figure 5:**
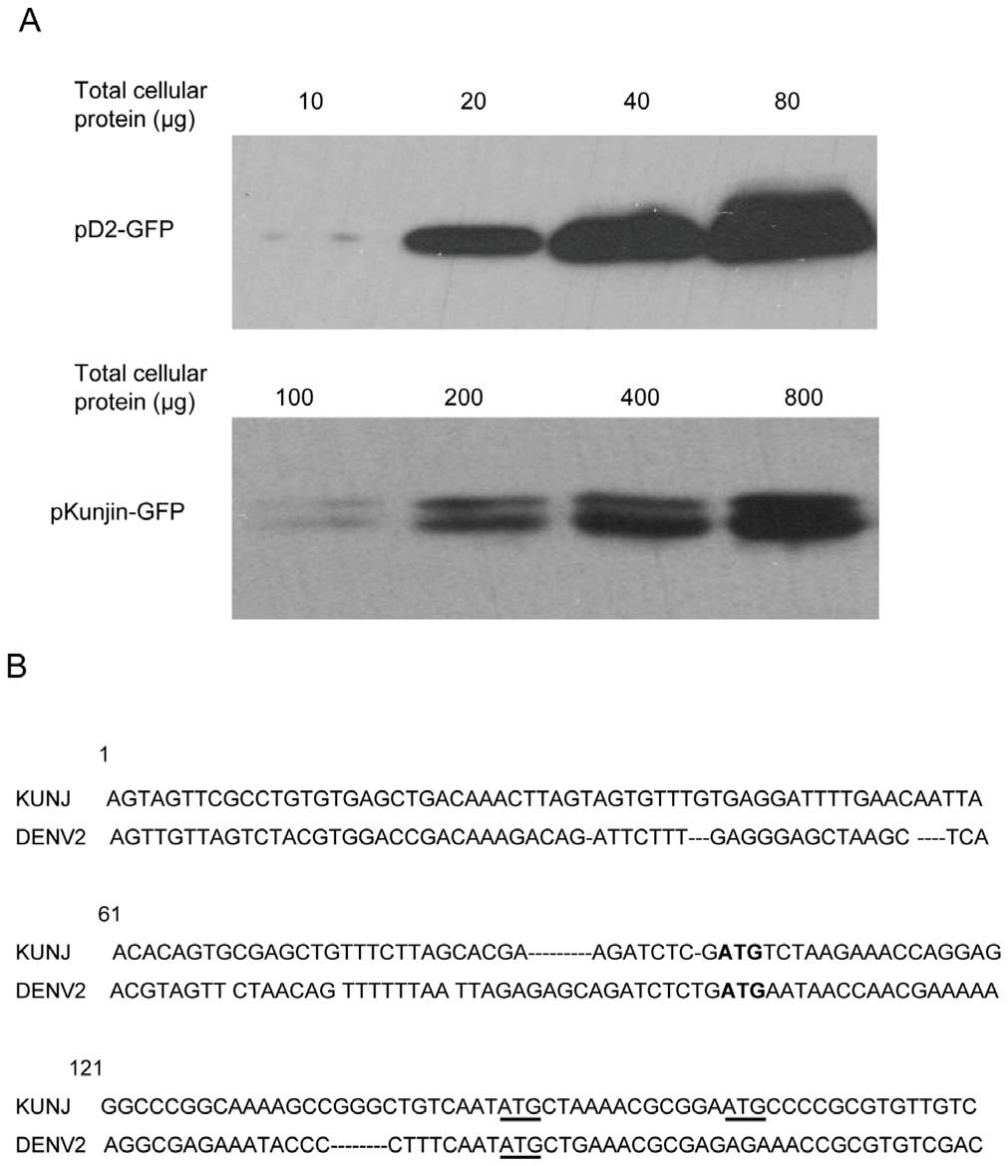
5′ cDNA sequence of Kunjin virus genome also contains an active cryptic bacterial promoter. (A) Western blots of cell lysates of *E. coli* cells transformed with pD2-GFP and pKUN-GFP probed with an anti-GFP antibody. (B) A comparison of cDNA sequences encoding the 5′ portion of the KUN and DENV2 genomes. The ATGs encoding the authentic viral initiation codons are shown in bold. The ATGs encoding codons utilised by *E. coli* for translation initiation are underlined.

Like DENV, Kunjin virus (KUN) is a mosquito-borne member of the flavivirus genus, and KUN cDNA clones are relatively more stable in *E. coli* than other flavivirus cDNA clones. The cDNA encoding mosquito-borne flavivirus 5′UTRs contain a T-rich region in a position analogous to the cryptic promoter described here for DENV2. Such T-rich regions might generally function as cryptic bacterial promoters of variable efficiencies, depending on their sequence similarity to the wild type bacterial promoter. To compare the efficiency of cryptic transcription in *E. coli* between flaviviruses with differing known cDNA stabilities, the pKUN-GFP plasmid was constructed by inserting cDNA encoding the 5′ 1–170 nt of the Kunjin virus (KUN) genome and the GFP sequence into a pUC18 vector. As before, the GFP sequence was placed in frame with the authentic viral initiation codon. Unlike *E. coli* cells transformed with pD2-GFP, cells transformed with pKUN-GFP did not fluoresce under UV light (data not shown). The amount of GFP protein detected by Western blot in *E. coli* cells transformed with pKUN-GFP was 10-fold lower than in cells transformed with pD2-GFP (Figure 5A). The amount of GFP mRNA detected by quantitative RT-PCR in *E. coli* transformed with pKUN-GFP was also 10-fold lower than the amount of DENV2 mRNA detected in *E. coli* transformed with pD2-GFP (Table 1). These data suggest that the cryptic promoter in KUN cDNA is less efficient than the DENV2 cryptic promoter, and confirm that cryptic transcriptional efficiency played the major role in controlling the amount of viral protein cryptically produced in transformed *E. coli*.

Interestingly, two bands were observed in Western blot analyses of lysates from pKUN-GFP transformed cells, while only a single band was observed from pD2-GFP transformed cell lysates. Cryptic transcription and subsequent translation of pKUN-GFP generated two GFP-containing products in *E. coli* that differed by approximately 500 Da (Figure 5A). A multiple sequence alignment of flavivirus C proteins showed that the Met residue whose codon is used by DENV2 as an initiation codon in bacteria is conserved in all other mosquito-borne flaviviruses and in all DENV serotypes [16] as part of a conserved NMLKR sequence motif. In KUN, this Met is encoded by 142AUG. An alignment of the KUN and DENV cDNA sequences used in this work are shown in Figure 5A. Unlike other mosquito borne flaviviruses, KUN and West Nile virus (WNV) contain a second Met residue, which is encoded by 157AUG. Translational initiation from the two internal Met codons would yield proteins that differed by 5 amino acid residues, which is consistent with the observation of two GFP-containing protein products in Western blots that differ by about 500 Da. By contrast, a protein product initiated from the authentic KUN start codon (97AUG) would be about 1500 Da or 2000 Da larger than proteins formed from 142AUG or 157AUG, respectively. Site directed mutagenesis of pKUN-GFP that converted 142AUG to 142UUG in the mRNA eliminated of the larger of the two protein products (data not shown), confirming that these products were formed by initiation from the two internal Met codons.

## Discussion

This study demonstrates that cryptic prokaryotic promoters exist in the cDNA coding for the 5′UTRs of the DENV2 and KUN genomes that are capable of directing spurious viral protein expression in *E. coli*. Similar cryptic promoters are likely to be present in most mosquito borne flaviviruses, because the cryptic promoter identified in the DENV 5′UTR cDNA resides in a T-rich region that is highly conserved among DENV serotypes and is common among members of the flavivirus genus. The 5′ UTR cDNA representing the other *Flaviviridae* genera, the pestiviruses and the hepaciviruses, do not contain similar T-rich promoter-like sequences and do not suffer the same instability as the flaviviruses. Hepatitis C virus, for example, can be cloned and propagated in *E. coli* using high copy number vectors [5]. Even within the flavivirus genus, the success of strategies to create full length infectious cDNA clones in *E. coli* has been highly variable. DENV cDNA clones are the most difficult among flaviviruses to construct. The DENV serotype 4 (DENV4) cDNA cloned into the pBR322 low-copy number plasmid [17] has been described as “metastable”, because bacterial colonies arise containing large deletions or insertions in the DENV4 sequence [9]. The pANCR low copy-number plasmid used to successfully establish infectious cDNA for WNV and JE [18] failed to produce full length infectious cDNA of DENV serotype 1 (DENV1) [10]. The reason for the differences in cloning difficulty among flaviviruses is unclear, but it has been speculated that DENV proteins are more toxic to *E. coli* than the viral proteins of other flaviviruses [10]. Alternatively, these differences might reflect the variable efficiencies of the cryptic promoters among the flavivirus cDNA. KUN and WNV full length cDNA, for example, are easier to clone in *E. coli* than full length DENV cDNA, which is consistent with our data showing that the cryptic promoter in the KUN 5′ UTR cDNA is less efficient in *E. coli* than the analogous cryptic promoter in DENV2 cDNA. All four DENV serotypes contain a sequence in the T-rich region between DENV nt 68 and 86 that could harbour a cryptic bacterial promoter. These sequences are similar but not identical, which might explain the subtle differences in cloning difficulty among Dengue viruses. A more extensive survey analysis of flavivirus cDNA clones would be required to fully understand the generality of the relationship between cryptic promoter efficiency and genetic stability of flaviviruses in *E. coli*.

Previous reports have shown that eukaryotic promoters, such as the cytomegalovirus (CMV) and the Rous sarcoma virus long terminal repeat (RSV LTR) promoters, are active in *E. coli*, and have postulated that leaky activity of these promoters may have contributed to transcription of viral sequences and subsequent viral protein expression [19], [20]. In most flavivirus cloning strategies, however, the T7 or SP6 phage promoters are employed instead, to allow *in vitro* transcription of viral RNA. Our data show that the T7 promoter in the vector carrying our reporter construct did not drive transcription in *E. coli*, and that the DENV 5′ UTR cDNA sequence was uniquely responsible for the observed downstream expression of virus derived protein sequence.

Because the DENV reporter constructs used in this study contained cDNA encoding only the first 170 nt of the DENV2 genome, additional cryptic promoters might exist further downstream in the full length DENV cDNA that could produce viral products contributing to toxicity in *E. coli*. However, early attempts to clone full length DENV2 cDNA in low copy number plasmids like pBR322 failed because of genetic instability when cDNA encoding the 5′ portion of the DENV2 RNA genome was present [21]. Attempts to clone infectious DENV4 cDNA into low copy bacterial vectors resulted in insertions and deletions in the E/NS1/NS2A region [9]. Stabilizing nonsense mutations were often found in the regions encoding structural proteins in constructs containing the cDNA of Japanese encephalitis virus (JEV) [11]. Thus, collective evidence suggests that the flavivirus structural proteins, which are located toward the 5′ end of the flavivirus ORF, contribute strongly to the observed toxicity in *E. coli*. It is reasonable to suggest that potential cryptic promoters in downstream cDNA might be less important for toxicity than cryptic promoters located in the 5′ UTR cDNA.

In addition to promoter activity, spurious expression of viral proteins in *E. coli* requires efficient translation initiation on the resultant mRNA. Prokaryotic translation normally utilises the interaction between the SD sequence in the 5′UTR of an mRNA and the anti-SD sequence in the 3′ end of the 16 S ribosomal RNA [22], [23]. However, non-canonical prokaryotic translation initiation mechanisms that are not dependent on the SD sequence have been reported. The ribosomal protein S1 (RPS1) can, for example, interact with an AU-rich sequence within 5′UTR to recruit and assemble the ribosomal initiation complex on mRNA containing short leader sequences lacking a viable SD sequence [24], [25]. Alternatively, the 70 S ribosomal complex can bind directly to leaderless mRNA and initiate translation [26]. Translation initiation on mRNAs derived from the DENV or KUN 5′UTR cDNA cryptic promoter, which possess a short 5′UTR of about 50 nt but lack a SD sequence, likely employs the RPS1 mechanism.

The utility of this work is to inform strategies for the effective and efficient cloning of full length infectious flavivirus cDNA. While the existing strategies that employ low copy number plasmids or *in vitro* genome assembly are useful workarounds, they do not address the root cause of the toxicity and genetic instability in *E. coli*. These measures are also not entirely satisfactory, as they are unwieldy and often produce low yields of metastable DNA. Simply mutating the cDNA in the DENV 5′ UTR region to knock out the cryptic promoter, however, would probably be unsuitable, as the flavivirus 5′ UTRs play important roles in virus genomic RNA replication and translation [15], [27], [28]. They share a high sequence homology and highly conserved RNA secondary structures, and mutations in the cDNA encoding the 5′ UTRs would likely be deleterious to virus replication efficiency. However, *E. coli* unexpectedly utilised a Met codon located downstream from the authentic DENV start codon to initiate translation, and a missense mutation at this codon completely abrogated protein expression from our reporter construct in *E. coli* while having no effect on protein expression in eukaryotic cells. This observation suggests that a similar mutation in the full length flavivirus cDNA might reduce or prevent spurious viral protein expression in *E. coli* and allow cDNA cloning of difficult or currently inaccessible flavivirus full length genomes. The contribution of downstream cryptic promoters to *E. coli* toxicity, if any, and the effect of mutating the highly conserved Met codon on virus viability have yet to be evaluated.

## Materials and Methods

### Mammalian and bacterial cell lines

BHK-21 clone 15 cells were cultured at 37°C in RPMI 1640 media (Gibco, USA) supplemented with 5% v/v foetal calf serum (FCS) and 100 U/ml penicillin, 100 µg/ml streptomycin and 2 mM glutamine (Gibco, USA) (growth medium). The data reported here were collected using competent *E. coli* DH5α cells (Promega, USA) for plasmid transformation and selected using ampicillin (100 µg/ml). The cells were cultured in LB media containing 100 µg/ml of ampicillin at 37°C.

### Plasmid Construction

#### pT7-D2-GFP

A dsDNA fragment containing the T7 promoter and DENV2 1–175 cDNA sequences was generated by PCR from the plasmid pGEM-D2-DI (GenBank access number: HM016517.1) using pfx DNA polymerase (Invitrogen, USA), and the D2-T7-5′UTR-NotI-F and D2-175-XbaI-R primers (Table S1). The fragment was then ligated into a pUC18 vector, which had been prepared by EcoRI and HindIII restriction digestion and by blunt ending using the Klenow fragment. The resultant plasmid, pT7-D2-175, contains a single SalI restriction enzyme site at nt 165–166 of the DENV2 sequence. A PCR product containing the GFP coding sequence flanked by SalI restriction sites was amplified from the pDSW208 plasmid [29] using the GFP-SalI-F and GFP-SalI-R primers (Table S1), and digested with the SalI restriction enzyme. The pT7-D2-175 plasmid was linearised by SalI restriction digestion. The pT7-D2-GFP construct was then constructed by inserting and ligating the GFP PCR product into the linearised pT7-D2-175 plasmid.

#### pD2-GFP, pT7-GFP, pΔ50D2-GFP, pΔ67D2-GFP and pΔ85D2-GFP

The D2-5′UTR-Not1-F, T7-GFP-Not1-F, D2-5′-51-Not1-F, D2-5′-68-Not1-F and D2-5′-86-Not1-F forward primers (Table S1) were used in individual PCR reactions with the common GFP-XbaI-R reverse primer (Table S1) to amplify deletion fragments from the pT7-D2-GFP template. The PCR products were digested with NotI and XbaI restriction enzymes to generate the appropriate overhanging sequences. The fragments were then ligated into a pUC-T7-D2-GFP plasmid that had been digested with the NotI and XbaI restriction enzymes.

#### pD2-GFP-74G, pD2-GFP-74GCG, pT7-D2-GFP-139T and pT7-D2-116TCC-GFP

Mutations of pD2-GFP and pT7-D2-GFP were made using QuickChange® Site-Directed Mutagenesis Kit (Stratagene, USA) following the manufacturer's instructions. The primers used for mutagenesis are listed in Table S1.

#### pKUN-GFP

A 1–173 nt cDNA sequence fragment of the Kunjin virus genome was amplified by PCR from the pAKUN/FLSDX2A plasmid [30] using the KUN-5′UTR-NotI-F and KUN-173-SalI-R primers (Table S1), and was digested with the NotI and SalI restriction enzymes. This fragment was ligated into the backbone of the pD2-GFP plasmid, which had been prepared by digestion with NotI and SalI restriction enzymes.

All plasmids were transformed into *E. coli* by heat shock followed by ampicillin selection on LB plates overnight at 37°C. Colonies were screened using colony PCR, and the inserts in the plasmids were confirmed by sequencing.

### Cell Imaging

*E. coli* was transformed with the indicated constructs and cultured overnight in LB medium at 37°C in the presence of ampicillin. A drop of cells was spread on a micro slide and fixed with 4% v/v paraformaldehyde in PBS buffer. The cells were gently washed twice with PBS and then mounted in 50% v/v glycerol-PBS. The images were obtained by florescence microscopy (Leica AF6000).

### RNA transcription and transfection

The plasmids pT7-D2-GFP and pT7-D2-GFP-139T were linearised by Xba1 restriction enzyme digestion and transcribed using the T7 MEGAscript transcription system (Ambion, USA). Transfection of BHK cells was performed by electroporating 5×10^6^ cells in 0.5 mL PBS buffer with 10 µg of *in vitro* transcribed RNA in a Gene Pulser XceII (Bio-Rad, USA) at 125 V, using 2 pulses of 25 ms each. Following electroporation, the cells were diluted in fresh growth medium and transferred to a 25 cm^2^ tissue culture flask at 37°C. The growth medium was replaced with fresh medium at 24 h post-electroporation and incubated for an additional 24 h. The cells were then lysed in 1 ml SDS-PAGE loading buffer prior to PAGE and western blotting.

### Western blot

*E. coli* cells were cultured for 24 h and harvested by centrifugation at 5000 g for 5 min. The cell pellets were lysed by heating in SDS-PAGE loading buffer at 95°C for 10 min. Where IPTG induction was employed, the overnight culture was diluted (1∶100) in fresh LB medium and grown at 37°C for 2 h prior to adding IPTG (final concentration 2 mM). The cells were incubated for a further 5 h at 37°C before harvesting.

Proteins from the cell lysates were resolved on 12% SDS-PAGE gels and transferred onto nitrocellulose membranes. The membranes were blocked with 4% v/v milk in PBS buffer at room temperature for 1 h and then probed with monoclonal anti-dengue capsid protein antibody (6F3.1) [13] or anti-GFP antibody (Sigma-Aldrich, USA). The peptide sequence determinant that is essential for epitope recognition by 6F3.1 was shown by octapeptide scanning to be^ 16^LKR^18^ [13], and is predicted to be present in all DENV2 protein products that initiate translation at either the canonical DENV start codon or the downstream in frame AUG. The bands were detected by enhanced chemiluminescence (Perkin Elmer) using species-specific secondary antibodies conjugated to horseradish peroxidase (DAKO, Denmark).

### Quantitative RT-PCR

Total RNA was extracted from *E. coli* bacteria using PureLink™ RNA Mini Kit (Invitrogen) following the manufacturer's instructions. RNA was reverse transcribed *in vitro* for 1 h at 50°C using Expand reverse transcriptase (Roche, Germany) using random oligonucleotide primers. Quantitative real-time PCR was performed on a Roter-Gene-6000 real-time PCR instrument (Corbett, Australia) using Light Cycle FastStart DNA Master Plus SYBR green I (Roche, Switzerland). Cycling conditions were 1 cycle of 95°C for 10 min followed by 35 cycles of 95°C for 10 sec, 58°C for 10 sec and 72°C for 25 sec. GFP RNA was quantified using primers SalI-GFP-F and GFP-362-R (Table S1). Results were normalized using the bacterial 16 S rRNA housekeeping gene that was amplified using the primers Bac-16S-F and Bac-16S-R (Table S1) as described elsewhere [31]. The results are reported in Table 1 as the log_10_ of the normalized copy number. Student's T test was used to compare the pKUN-GFP and the pD2-GFP mutant constructs with the unmodified pD2-GFP.

## Supporting Information

Table S1 Complete list of primer sequences used for amplification or site directed mutagenesis.
(DOCX)
