# Supplementary material for: Identification of a Cryptic Prokaryotic Promoter within the cDNA Encoding the 5′ End of Dengue Virus RNA Genome
Source: PLoS One. 2011 Mar 31;6(3):e18197. doi: 10.1371/journal.pone.0018197 (PMC3069047; doi:10.1371/journal.pone.0018197)
Supplement: Table S1 — Complete list of primer sequences used for amplification or site directed mutagenesis. (DOCX) [file pone.0018197.s001.docx]

Table S1. Primer sequences

| Primer name | Primer sequence |
| --- | --- |
| D2-T7-5’UTR-Not-F | 5’GCGGCCGCGTAATACGACTCACTATAGGAGTTGTTAGTCTACGTGGACCG 3’ |
| D2-175-XbaI-R | 5’TCTAGATCACAGTCGACACGCGGTTTCTC 3’ |
| Sal I-GFP-F | 5’GTGTCGACTGTGAGCAAGGGCGAGGAGCTGTTC 3’ |
| Sal I-GFP-R | 5’CAGTCGACTCACTTGTACAGCTCGTCCATGGC 3’ |
| T7-GFP-NotI-F | 5’TTGCGGCCGCGTAATACGACTCACTATAGGATGGTGAGCAAGGGCGAGGAG 3’ |
| GFP-XbaI-R | 5’ CTCTAGATCACTTGTACAGCTCGTCCATGC 3’ |
| D2-5’UTR-NotI-F | 5’TTGCGGCCGCAGTTGTTAGTCTACGTGGAC 3’ |
| D2 139A-to-T-F | 5’GCCTTTCAATTTGCTGAAACGCG 3’ |
| D2-139a-TO-t-R | 5’CGCGTTTCAGCAAATTGAAAGGC 3’ |
| D2-5’-51-NotI-F | 5’ATGCGGCCGCTCAACGTAGTTCTAACAG 3’ |
| D2-5’-68-NotI-F | 5’ATGCGGCCGCAGTTTTTTAATTAGAGAGCAG 3’ |
| D2-5’-86-NotI-F | 5’ATGCGGCCGCAGATCTCTGATGAATAACC 3’ |
| D2-116AGG-TCC-F | 5’GAATAACCAACGGAAAATCCCGAAAAACACGCCTTTC3’ |
| D2-116AGG-TCC-R | 5’GAAAGGCGTGTTTTTCGGGATTTTCCGTTGGTTATTC 3’ |
| KUN-5’UTR-Not1-F | 5’ TGCGGCCGCAGTAGTTCGCCTGTGTGAGCTG 3’ |
| KUN-173-Sal1-R | 5’TGTCGACAACACGCGGGGCATTCC 3’ |
| D2-74-G-F | 5’GTTCTAACAGTTTGTTAATTAGAGAGCAG 3’ |
| D2-74-G-R | 5’CTGCTCTCTAATTAACAAACTGTTAGAAC 3’ |
| D2-74-TTT-GCG-F | 5’GTTCTAACAGTTTGCGAATTAGAGAG CAG 3’ |
| D2-74-TTT-GCG-R: | 5’CTGCTCTCTAATTCGCAAACTGTTAGAAC 3’ |
| Bac-16S-F | 5’GCGGTTTGTTAAGTCAGATGTG 3’ |
| Bac-16S-R | 5’GACTCAAGCTTGCCAGTATCAG 3’ |
